# Supplementary material for: Causal Relationship between Aging and Anorexia Nervosa: A White-Matter-Microstructure-Mediated Mendelian Randomization Analysis
Source: Biomedicines. 2024 Aug 16;12(8):1874. doi: 10.3390/biomedicines12081874 (PMC11351342; doi:10.3390/biomedicines12081874)
Supplement: Supplementary file 1 [file biomedicines-12-01874-s001.zip › Supplementary Figure/Figure S8/Leaveoneout plot of MR analysis for TL to ubm-b-1493.pdf]

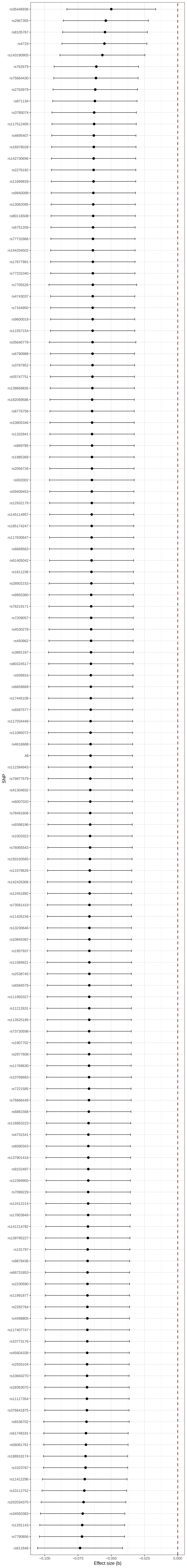
rs35446936
rs2967355
rs8105767
rs4724
rs143190905
rs762679
rs75664430
rs2763979
rs871134
rs3785074
rs117512405
rs4695407
rs16978028
rs142730696
rs2276182
rs11699829
rs9940099
rs13062095
rs80116508
rs6751209
rs77732866
rs144204502
rs17677991
rs77231040
rs7705526
rs4743037
rs7164950
rs9600019
rs11557154
rs35640778
rs6790988
rs3767952
rs55747751
rs139669835
rs182059586
rs6776756
rs10805346
rs1332941
rs869785
rs1985369
rs2056726
rs932002
rs59409453
rs12932179
rs145114957
rs185174247
rs117630647
rs6669563
rs61405042
rs1611236
rs28502153
rs9955360
rs76219171
rs7209057
rs4530278
rs450962
rs3891167
rs80324517
rs939916
rs6659669
rs17445108
rs6587577
rs117034449
rs11085072
rs4616688
All
rs112394943
rs79977579
rs41304832
rs6007020
rs78491606
rs9398196
rs1003322
rs76065543
rs150150565
rs11579626
rs142426306
rs12451892
rs73581419
rs11426156
rs13230646
rs10845387
rs1957937
rs11584821
rs2538745
rs6584579
rs111950327
rs11212631
rs113525195
rs73730598
rs1907702
rs2977608
rs11769630
rs10768863
rs7221585
rs76666449
rs6881568
rs116863223
rs4731541
rs6590343
rs137901416
rs8102497
rs12369950
rs7099229
rs12412214
rs17803849
rs141214782
rs139795227
rs131797
rs9878436
rs66731853
rs2230590
rs11991877
rs2282764
rs4498805
rs117407747
rs10773176
rs45604339
rs2555104
rs10840270
rs28363070
rs11117354
rs376641875
rs6536702
rs61748181
rs56061761
rs188918174
rs1023767
rs11412296
rs10112752
rs202034370
rs34550383
rs1291143
rs7790856
rs611646
SNP
-0.100
-0.075
-0.050
-0.025
0.000
Effect size (b)
